# Determining optimum plant density and nitrogen rate using field experiment and model simulation

**DOI:** 10.1038/s41598-025-95862-6

**Published:** 2025-07-18

**Authors:** Bizuwork Tafes Desta, Sisay Eshetu Tesema, Gebrekidan Feleke Mekuria, Almaz Meseret Gezahegn, Alemayehu Zemede Lemma

**Affiliations:** Debre Zeit Center, Ethiopian Institute of Agricultural Research, P. O. Box 32, Debre Zeit, Ethiopia

**Keywords:** Durum wheat, Nitrogen fertilizer, Plant density, Seasonal analysis, Ecology, Physiology, Plant sciences

## Abstract

Poor crop management practices are key factors leading to a significant reduction in durum wheat yield in the central highlands of Ethiopia. The aim of this study was to determine optimum plant density and nitrogen rate that increase durum wheat productivity while reducing environmental impacts. A combination of data from field experiments conducted from 2017 to 2020 under rainfed conditions and simulation data of CERES-Wheat model were used for this study. The CERES-Wheat model was calibrated for Utuba cultivar from 3-years (2017–2019) field experiment data. The model was further evaluated with the experimental data conducted during the 2020 cropping season under four plant densities and four nitrogen fertilizer rates. Because of differences in temperature and rainfall patterns during the potential growing season, seasonal analysis was used to determine the optimum plant density and N rate using 37 years (1985–2022) of historical weather data. The simulation results suggested that 275 plants m^−2^ with an application of 200 kg ha^−1^ N increased grain yield, improved nitrogen use, and produced the highest economic return while minimizing environmental risk under rainfed conditions. Compared with the current plant density (175 plants m^−2^) and N fertilizer (100 kg ha^−1^) an increase in plant density to 275 plants m⁻^2^ with 200 kg ha⁻^1^ N resulted in a 49% increase in grain yield by about 49%, N use efficiency by 23% with the highest net return (2114 US$ ha^−1^). In general, this study showed that the CERES-Wheat model can be a promising tool for providing crop management recommendations under rainfed durum wheat farming.

## Introduction

The expansion of agricultural land to the marginal area was the primary factor to increase food production, which resulted into GDP growth in the central highlands of Ethiopia since the early 2005^1^. However, this horizontal increase in farmland is facing various constraints that hamper crop productivity. Severe land degradation, poor inherent fertility, high dependence on rainfall, and low uses of plant density and fertilizers by smallholder farmers were among the major constraints that could be mentioned^2,3^. In addition, owing to the rapidly growing population in the rural parts of Ethiopia, particularly in the central highlands of the country, the size of cropland per capita has been decreasing drastically. For instance, in Oromia, which is less populated, the average farm size is 1.2 ha, whereas in the densely populated Southern Nations, Nationalities, and Peoples’ Region (SNNPR), the average farm size is 0.5 ha^4^. This indicates that smallholder farming systems will continue to dominate the agriculture sector and the average farm sizes will continue to decline because the further expansion of cropland will become more difficult, while the population will continue to increase^5^. Therefore, future agricultural development should focus on raising productivity per unit area instead of expanding the cultivated area.

Sustainable intensification is understood to encompass a wide range of practices, including appropriate but not limited to conservation agriculture, with the potential to produce more food from the existing agricultural land in a valuable and changed environment while maintaining resources^6^. Agronomic intensification practices, including plant density and nutrient supply, have the potential to enhance agricultural production outputs^7^. Integrated management strategies, such as optimum plant density and soil nutrient application, achieve the highest yield, adulate grain quality, and long-term sustainability based on environmental circumstances^8,9^. For instance, the supply of optimum nitrogen fertilizer and plant density have been proven to be keyways to increase crop yields in dryland farming^10–13^. In the central highlands of Ethiopia, an increase in nitrogen fertilizer from 100 to 150 kg ha^−1^ in combination with the optimum plant density (approximately 200 plants m^−2^) increased bread wheat yield from 2534 to 3567 kg ha^−1^ in^14^ showed a 29% increase in wheat yield. In another study conducted in the northern part of Ethiopia, durum wheat grain yield showed a quadratic response to nitrogen fertilizer, and the maximum grain yield (5182 kg ha^−1^) was obtained at 270 kg ha^−1^ N, with the highest net benefit^15^. These findings confirm that the use of the optimum nitrogen fertilizer rate significantly increased durum wheat yield^16^ also suggested a possible approach to increasing wheat grain yield per unit of land by adjusting nitrogen management along with other crop practices.

Plant density is an important agronomic intensification parameter. Plant density has a significant positive effect on wheat yield^17,18^reported a positive linear improvement in grain yield in durum wheat at rates as high as 450 plants m^−2^, which was more than twice the rate of standard practices (210 plants m^−2^) at that time. This positive association between plant density and durum wheat yields was also reported by^16,19^. In addition to increasing grain yield, increasing plant density, improving nitrogen utilization and uptake efficiency, and increasing wheat root length and density, overall increases below and above ground plant demand^20^. In Ethiopia, most farmers cultivate durum wheat using low plant density and insufficient nitrogen fertilizer. In practice, a low plant density of 175 plants m^−2^ and blanket application of nitrogen fertilizer (approximately 100 kg ha^−1^) restrict yield and nitrogen use efficiency^18,21^. In contrast, durum wheat grain yields as high as 5670 kg ha^−1^ have been achieved under high plant densities (225 plants m^−2^) and nitrogen fertilizer rates (150–200 kg ha^−1^) in Ethiopia under irrigation conditions^22^. Appropriate plant densities, combined with optimum nitrogen management, are likely to increase grain yield and nitrogen use efficiency^23^. However, in the central highlands of Ethiopia, there is limited evidence on the interaction between nitrogen fertilization and plant density. Specifically, there is no documented research on the combined effects of plant population and nitrogen fertilizer rates on durum wheat yields under rainfed conditions. Understanding these interactions is essential for enhancing and sustaining crop productivity in this region. Thus, knowledge of the interaction between nitrogen nutrients and plant density is important for increasing and maintaining crop yields in the central highlands of Ethiopia.

Although field-based experiments are usually effective, they are time-consuming, require expensive resources, and take a longer time to draw valid recommendations. Thus, process-based models have proven to be useful tools for investigating the impact of climate variability and change on crop productivity, resource use efficiency, and environmental impacts on the agricultural system^19^. Moreover, the application of a process-based model also provides an option for designing climate-resilient management strategies to tackle the enormous challenges facing agricultural productivity and to generate knowledge for aiding agricultural developments that would otherwise be impossible through field experimentation^24^.

The CERES-Wheat model, which is embedded in DSSAT, is widely used to determine the best strategic plant density, irrigation management, and nutrient dynamics^7,22,25,26^. However, no study has used combined experimental and model simulation approaches of plant density and nitrogen fertilizer rate interaction on the long-term impact of water limitation on durum wheat yield and economic values. Moreover, research on plant density and nitrogen fertilizer for durum wheat in Ethiopia began in the late 1960s in the central highlands^27^. Typical recommended practices for durum wheat in the central highlands of Ethiopia are 175 plants m^−2^ and 150 kg ha^−1^ N^28,29^. Despite previous studies, there is still a lack of systematic investigation on how plant density and nitrogen fertilizer rate interact with the long-term impact of water-limited conditions on durum wheat yield and economic value. In addition, most of the plant density and nitrogen fertilizer recommendations for durum wheat are outdated, and no longer reflect the current agro-climatic variability and change. To fill this gap, we carried out a study that combines experiments and modeling at the field level for durum wheat in central highlands and covering a wider range of weather conditions. The following objectives were addressed: (i) to calibrate the CERES-Wheat model for durum wheat cultivar, (ii) to evaluate the performance of the CERES-Wheat model to simulate the growth, development, and yield of durum wheat under rain-fed cultivation, and (iii) to apply the CERES-Wheat model to determine the optimum plant density and nitrogen fertilizer supply using long-term historical daily weather data for durum wheat rain-fed cultivation.

## Materials and Methods

### Description of the study area

The experiments were conducted over four consecutive years (2017, 2018, 2019, and 2020) and the main wheat cropping season (June–November) at Memir Hager (8°46′33.5″ N and 3916′40.7″ E). It is found in the Minjar Shenkora district in the Amhara region of Ethiopia. The area has a typical wet-dry climate, with a monsoon season (rain) and summers typically running from June to August. Spring is from September to November, which is the harvest season in the area. The cold, dry season in the area, spanning from December to February, is characterized by cooler temperatures compared to other seasons and is recognized as the dry period. Autumn, which occurs from March to May, marks the agricultural land preparation season in the region.

### Soil characteristics and weather conditions of the experimental area

The soil’s physical, chemical, and hydraulic properties (field capacity, permeant wilting point, bulk density, saturated water), texture, pH, cation exchange capacity (CEC), ammonium and nitrate concentrations, and soil organic matter were obtained from the soil profile description from the Global High-Resolution Soil Profile Database for crop modeling application^30^, based on the latitude and longitude of its geographic grid (Table 1).

**Table 1:** Description of soil profile properties (lower soil water limit (LSL), drained soil water upper limit (DUL), saturated upper limit (SAL), bulk density (BD), organic carbon (OC), clay content (SCL), silt content of the soil (SSI), total N (TN), soil phosphorous (P), soil pH (pH) and soil cation exchange capacity (CEC) of the soil used in CERES-wheat model simulations. LSL, DUL, and SAL were calculated using DSSAT 4.8.2.

Depth (cm)	LSL	SDUL	SSAT	BD	OC	SCL	SSI	TN	pH	CEC (Cmol-kg^−1^)
	(cm^3^ cm^−3^)			g °cm^−3^	(g kg^−1^)					
5	0.213	0.34	0.430	1.18	2.87	35.6	27.2	0.12	6.4	35.7
15	0.224	0.35	0.436	1.20	2.43	37.5	26.5	0.09	6.5	31.3
30	0.239	0.37	0.444	1.23	1.85	40.1	25.3	0.07	6.6	30.4
60	0.253	0.38	0.452	1.28	1.18	42.5	24.2	0.06	6.7	31.7
100	0.253	0.38	0.451	1.34	0.69	42.4	23.7	0.05	6.8	31.9
200	0.244	0.37	0.444	1.40	0.39	41.0	23.2	0.05	7.0	31.8

**Fig. 1:**
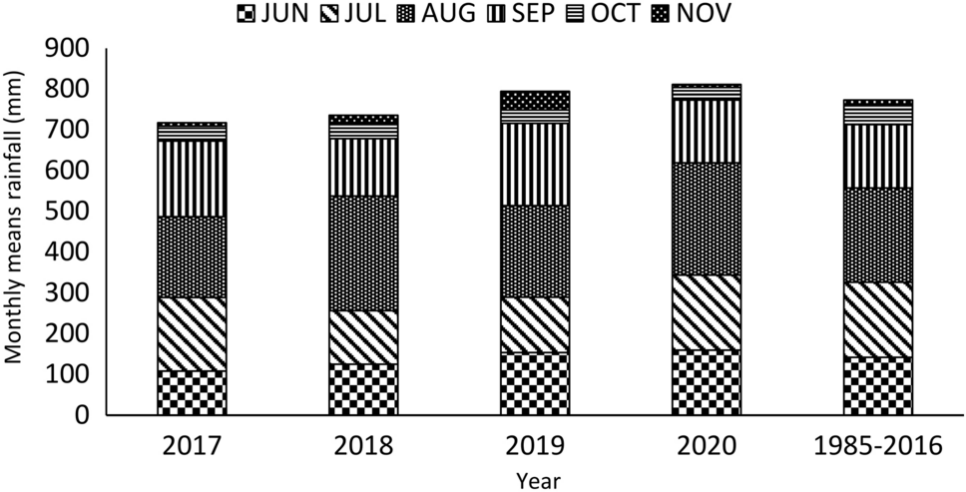
Average monthly rainfall of 33 years (1985–2016) and monthly means rainfall of 2017, 2018, 2019, and 2020 of wheat cropping months (June–November) of the experimental area.

**Fig. 2:**
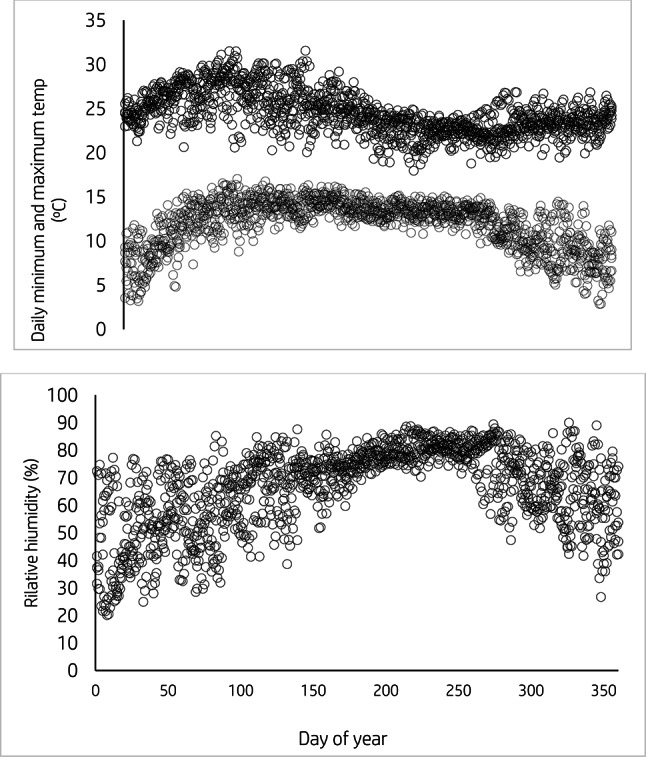
Daily maximum temperature (top graph), daily minimum temperature (center graph), and daily relative humidity (bottom graph) during the study period between (2017 and 2020) of the experimental area.

**Fig. 3:**
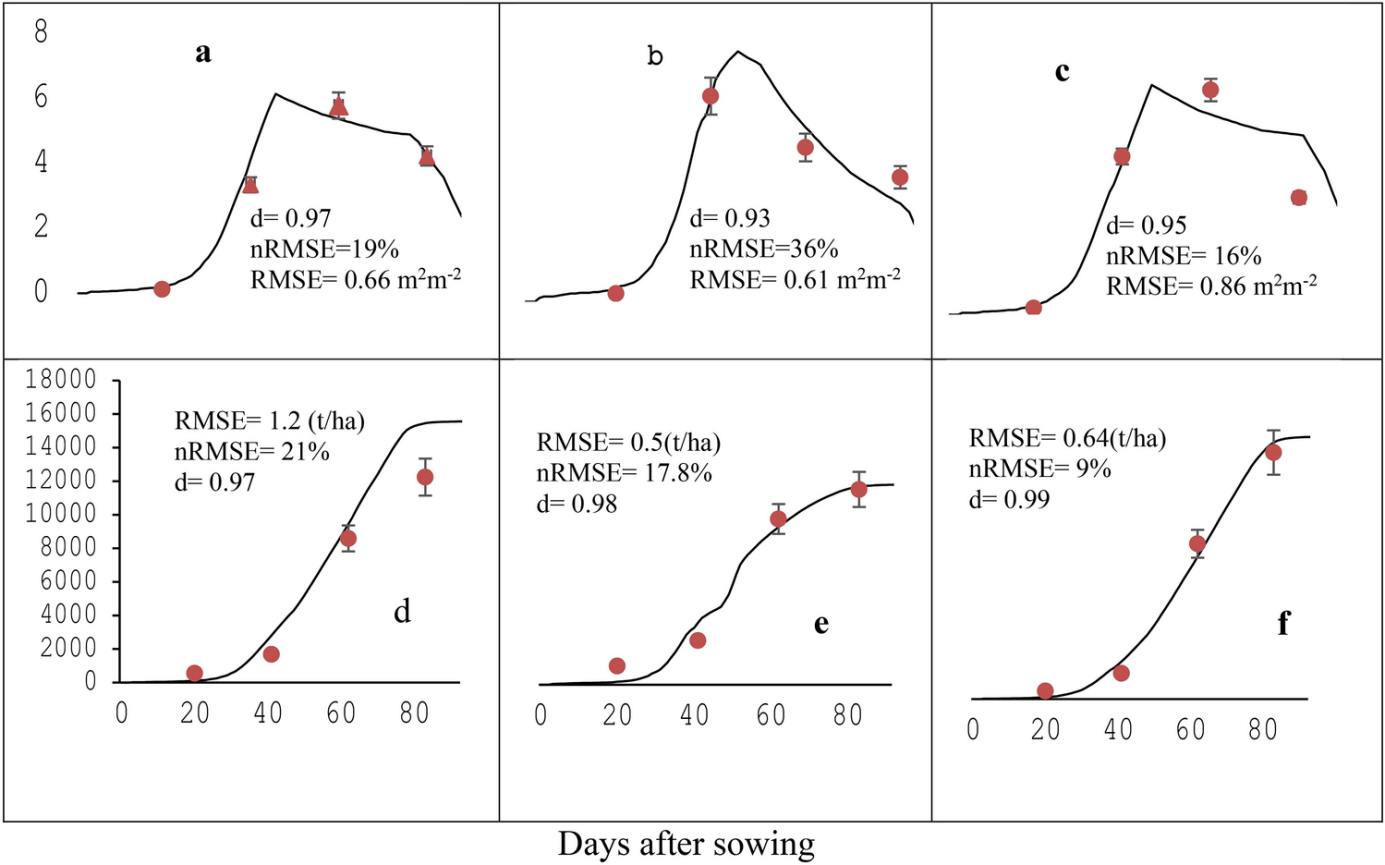
Simulated (continuous lines) and measured (circular with error bar symbols) leaf area index (**a** 2017, **b** 2018, **c** 2019) and top weight (**d** 2017, **e** 2018, **f** 2019) for durum wheat variety Utuba at optimum plant density (225 plants m^−2^ and N fertilizer (200 kg ha^−1^) at Memir Hager, used for model calibration.

Historical weather data for daily maximum and minimum air temperature, solar radiation, relative humidity, rainfall, and wind speed from 1985 to 2022 were obtained from the Ethiopian National Meteorology Agency (NMA) and used for long-term simulation analysis. From 2017 to 2020, daily weather data, including maximum and minimum air temperature, rainfall, relative humidity, solar radiation, and wind speeds, were obtained from a local weather station (Minjar Shenkora District Meteorological Station), which was near the experimental sites and used for model calibration and evaluation. Figure 1 presents the 31-year average monthly rainfall data (1985–2016) alongside the monthly mean rainfall for the years 2017, 2018, 2019, and 2020 during the durum wheat cropping months at the experimental site. The daily maximum air temperature, daily minimum air temperature, and relative humidity during the experimental periods (2017, 2018, 2019, and 2020) are illustrated in Figs. 2 and 3, respectively.

### Experimental set-up and field management

Experiments conducted from 2017 to 2019 were used for model calibration. The durum wheat cultivar ‘Utuba’ at an optimum planting density (225 plants m^−2^), nitrogen (urea at a rate of 200 kg ha^−1^), and phosphorus (di-ammonium phosphate) 18% N and 46% P_2_O_5_) at a rate of 100 kg ha^−1^ was planted on July 27, 2017, July 26, 2018, and July 28, 2019. The plot size was 5 m x 5 m (25 m^2^) and was replicated three times. The experiment undertaken during the 2020 cropping season was used for the model evaluation. The experiment included the following treatment combinations: four planting densities: 175 plants m^−2^ (PD_1_), 225 plants m^−2^ (PD_2_), 275 plants m^−2^ (PD_3_), and 325 plants m^−2^ (PD_4_) of the Utuba cultivar, and four N fertilizer rates: 0 (control) N_0=0_, 100 (N_1=46_), 150 (N_2=69_), and 200 (N_3=92_) kg ha^−1^ urea fertilizer. For phosphorus fertilization, 100 kg/ha of triple superphosphate (TSP) was applied, providing 46 kg/ha of P₂O₅ as the phosphorus source*.* The treatments were laid down in a randomized complete block design (RCBD) in a factorial arrangement, and each treatment was replicated three times. The plot size was 3 m × 3 m (9 m^2^) and consisted of 16 rows, spaced 20 cm apart. The net central areas of each plot, consisting of 12 central rows of 2.80 m length were used for data collection. The seeds were sown with a row spacing of 20 cm using a hand drill. For nitrogen fertilizer, two split applications, the first one-third N fertilizer was applied at planting, and the remaining (two-thirds N) dose was applied 21 days after crop planting. A full dose of phosphorus fertilizer was applied uniformly at planting. All other crop management practices, such as weeding, diseases, and insect pest protection, were uniformly implemented for all treatments.

### Trait measurements

Wheat phenology traits, such as anthesis and maturity date, were defined by the plant and showed visual signs of the stage being considered. The anthesis date was recorded when approximately 50% of the plants in a plot produced spikes. The maturity date was obtained when the leaves and vegetative parts of the crop were light yellow in color. In the season, the top weight of the plant was determined based on a randomly selected area of 0.25 m^2^ sample harvested at four Zadoks growth (GS24 (tillering), GS40 (booting), GS60 (flowering), and GS80 (grain filling) stages of each plot and dried in an oven at 70 °C for 48 h, and their weights were determined using an electronic balance. The leaf area index was measured using a plant canopy imager (Model CI-110), taking readings from the middle eight of the 16 rows per plot in all replications by passing the instrument on the top of the canopy at a height of 60 cm. Readings were taken at four growth stages, (GS24 (tillering), GS40 (booting), GS60 (flowering), and GS80 (grain filling). The crop was harvested manually from four central rows 2 m in length in each row to determine the final top weight, grain yield (GY), and harvest index. After harvesting, the threshed grains were separated, cleaned, and weighed using an electronic balance. GY was adjusted to a moisture content of 12.5% wet bases, and a moisture tester was used to test the moisture content of the grain. The Harvest index was computed using the formula of^31^.

$${\text{HI}}\, = \,{\text{GY}}/{\text{top weight}}$$

### Model calibration and evaluation

The CERES-Wheat model embedded in Decision Support Systems for Agrotechnology Transfer-DSSAT v4.8.2^32^ was used in this study. The cultivar Utuba coefficients of the model were calibrated using data collected from the optimum inputs treated experiments conducted during three growing seasons from 2017, 2018, and 2019. Genetic coefficients were calibrated using the procedure described by^7^. Adjustments were made sequentially, starting with phenological (anthesis and maturity date) traits and crop growth (leaf area index (LAI), top weight, and GY) parameters. The GLUE coefficient estimator was used for parameter estimation. In total, 10,000 random parameter sets were generated using an independent dataset for the crop-growing seasons. After calibrating the cultivar coefficients, the accuracy of the model was evaluated using experimental data collected in 2020. The observed data, such as in-season growth, LAI, and top weight, at final harvest biomass, GY, and harvest index, were collected and compared with simulated values of across each plant densities and nitrogen levels.

### Statistical analysis

For both calibration and evaluation, the results were checked using different statistical indices, including the normalized root-mean-square error (RMSE), normalized RMSE (nRMSE), index of agreement (d), and model efficiency (E). The statistical indices are described as follows.

1 $$\text{RMSE }=\sqrt{\frac{{\sum }_{i=1}^{n}{\left({s}_{i}-{M}_{i}\right)}^{2}}{n}}$$

2 $$\text{nRMSE }= \frac{ RMSE}{\overline{M} }$$

3 $$\text{E }=1-\frac{{\sum }_{i-1}^{n}{\left({S}_{i}-{M}_{i}\right)}^{2}}{{\sum }_{i=1}^{n}{\left({M}_{i}-\overline{M }\right)}^{2}}$$

4 $$\text{d }=1-\frac{{\sum }_{i=1}^{n}{\left(S-{M}_{i}\right)}^{2}}{{\sum }_{i=1}^{n}{\left(\left|{S}_{i}-{M}_{i}\right|+\left|{M}_{i}-{\overline{M} }_{i}\right|\right)}^{2}}$$

where n is the total number of datasets measured, and simulation values and M is the average of the measured values. The normalized RMSE (nRMSE) provides a measure (%) of the relative difference between the measured and simulated data. It is generally agreed that the model performance is excellent when the nRMSE value is less than 10%, good when the nRMSE is between 10 and 20%, fair when the nRMSE is between 20 and 30%, and poor when the nRMSE is greater than 30%^33,34^.

### Model application

After the calibration and evaluation procedures were completed, the model was used to simulate grain yield, agronomic efficiency, and economic value of treatments using seasonal analysis programs (DSSAT v4.8.2^32^. The simulation used a 37-year daily weather data from 1985 to 2022. A total of 48 combination comprised of six plant density and eight nitrogen fertilizer rates, were employed for the simulation. The plant densities ranged from 175 to 425 plants m^−2^ at an interval of 50 plants m^−2^ and N rate treatments ranged from 0 to 350 kg nitrogen ha^−1^ in an interval of 50 kg ha^−1^ were used for simulation. Following the simulation, the outputs were processed using the biophysical and economic analysis options of the program. These analyses and comparisons can identify and quantify the variability in crop performance associated with the interaction between weather and soil factors in the physical environment.

Agronomic efficiency (AE) calculated as the ratio of the difference in grain yield with and without nitrogen application divided by the total applied^35^.

5 $$\text{Agronomic efficiency }(\text{AE},\text{ kg kg}-1) = \frac{{Y}_{N }-{Y}_{O}}{{A}_{N}}$$

where Y_N_ is the grain yield from treatments with nitrogen fertilizer, Y_0_ is the grain yield without N fertilizer treatment, A_N_ is the amount of N fertilizer applied.

#### Economic analysis

The results of each combination of plant density (175 (PD_1_), 225 (PD_2_), 275 (PD_3_), 325 (PD_4_), 375 (PD_5_), and 425 (PD_6_) plants m^−2^) and nitrogen fertilizer rates of 0 (control) (N_0_), 50 (N_1_), 100 (N_2_), 150 (N_3_), 200 (N_4_), 250 (N_5_), 300 (N_6_), and 350 (N_7_) kg ha^−1^ were also evaluated for economic feasibility using the mean–Gini dominance analysis^36^. The evaluation procedure of the seasonal analysis program calculates the monetary return for each treatment based on the highest economic return Gini coefficient (GC). The gross margin (US$ t^−1^) for each combination of treatments was determined using the following equation:

$${\text{GM}} = {\text{ Y }} \times {\text{ P - N }} \times {\text{ C - V}}$$

where GM is the gross margin, Y is the simulated durum wheat grain yield (kg ha^−1^), P is the price of durum wheat (545.23 US$ kg^−1^) average of the last four years, 2017, 2018, 2019, and 2020), N = nitrogen application rate (kg ha^−1^) per treatment, C = the cost of nitrogen fertilizer (0.82US $kg^−1^), and V is the base production cost (887.00US $ha^−1^). The base production cost, durum wheat grain, and nitrogen fertilizer prices were obtained from the Economic Survey of the Debre Zeit Agriculture Research Center of Ethiopia (unpublished data).

##### Result and discussion

### Model calibration

The genetic coefficients of Utuba were calibrated using the GLUE method. The values of the seven genetic coefficients that determined the vegetative and growth stages (P1D, P1D, P5, and PHINT), and grain characteristics (G1, G2, and G3) are presented in Table (2). The genetic coefficients were estimated sequentially: first vegetative, followed by grain characteristics, using 10,000 rounds of the GLUE method^7,37^ used the GLUE method to accurately estimate the genetic coefficients of the DSSAT-CERES-Wheat and Maize models for winter wheat production in Beijing, China, and for sweet corn production in northern Florida, USA, respectively.

**Table 2:** Calibrated genetic coefficient of *Utuba* used with CERES-Wheat model.

Cultivar traits	Genetic coefficient	Unit	Value
Days, optimum vernalization temperature, required for completed vernalization	P1V	d	0.02
Photoperiod response (% reduction in rate/ 10 h drop in photoperiod	P1D	%	70.7
Grain filling (excluding lag) phase duration	P5	°C. d	577
Kernel number per unit canopy weight at anthesis	G1	no. g^−1^	32
Standard kernel size under optimum condition	G2	mg d^−1^	33
Standard, non-stressed mature tiller weight (milligram)	G3	g	4.1
Thermal time between successive leaf tip appearances	PHINT	°C d	115

### Durum wheat phenology

The calibrated CERES-Wheat model accurately simulated durum wheat phenology (anthesis and maturity date), and the values are presented in Table 3. The model predicted the dates from sowing to anthesis with a 0 (zero) difference and dates from sowing to maturity with a difference of 2 days (107 and 109 dates) between the simulated and measured values (Table 3). The four statistical indicators via root mean error square (RMSE), normalized RMSE (nRMSE), model efficiency (E), and index of agreement (d) were used to evaluate the simulated and measured values of the anthesis and maturity dates. The statistics were indicated in an excellent agreement, with RMSE, nRMSE, E, and d values of 1.00 days, 1.61%, 0.92, 0.85 and 1.41 days, 1.31%, 0.99 and 0.91, respectively (Table 3). Similarly, a close agreement was observed between the simulated and observed anthesis and maturity dates^22,38^. The model simulated anthesis and aturity dates with RMSE and nRMSE lower than 2 and d values greater than 0.85, respectively, for wheat in Ethiopia. In general, their results confirmed that the calibrated DSSAT-CERES-wheat model is appropriate and convenient for the anthesis and maturity dates of durum wheat in the central highlands of Ethiopia.

**Table 3:** Statistical evaluation of simulated (S) and observed (O), root-mean-square error (RMSE), normalized RMSE (NRMSE), model efficiency (E) and index of agreement (d) value for phenology (anthesis and maturity date) and leaf area index at (grain filling stage) and top weight, harvest index, and grain yield at final harvest for model calibration of durum wheat cultivar, Utuba (n = 3).

Variable	S	O	E	RMSE	NRMSE	d
Anthesis date	62	62	0.92	1.00	1.61	0.85
Maturity date	109	107	0.99	1.41	1.31	0.91
Grain yield (10^3^ kg ha^−1^)	4346	4373	0.98	0.50	11.50	0.89
Harvest index (%)	0.38	0.37	0.94	0.02	5.40	0.98
LAI (m^2^ m^−2^)	4.8	5.1	0.86	0.57	11.18	0.95
Top weight (10^3^ kg ha^−1^)	12,296	12,695	0.92	1.56	12.31	0.96

LAI: leaf area index.

### Leaf area index and top weight

**Fig. 4:**
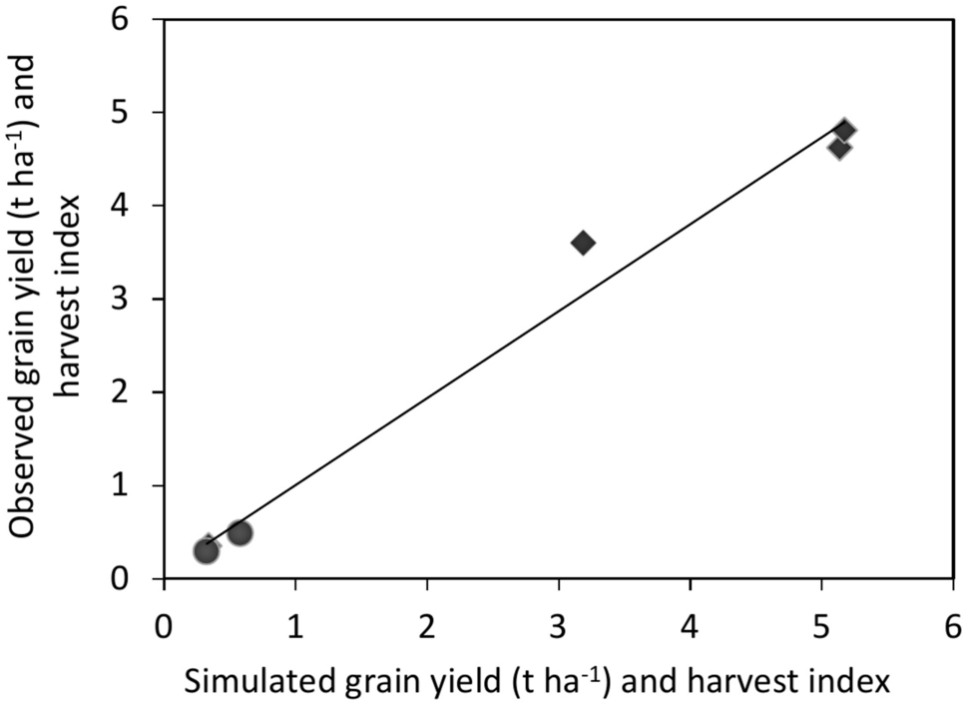
Comparison of simulated and measured grain yield (square symbol) and harvest index (circle symbol). Data from 2017, 2018, and 2019 were used to calibrate the model.

The simulated and measured values of LAI and top weight at four Zadoks growth stages (GS24 (tillering), GS40 (booting), GS60 (flowering) and GS80 (grain filling) during the experimental years of 2017, 2018 and 2019 were considered satisfactory because all results were within the acceptable range of statistics with the value of RMSE ranged from 0.48 to 0.66 m^2^ m^−2^, nRMSE from 19 to 27%, d value ranged from 0.93 to 97 for LAI (Fig. 3 A, B, C). The value of RMSE for top weight ranged from 582 to 1228 kg ha^−1^, the value of nRMSE ranged from 9.4 to 21.2%, the E value ranged from 0.96 to 0.98 and the d value ranged from 0.98 to 0.99 (Fig. 4 D, E, F). The simulation quality of LAI for the durum wheat growth stages was well matched for 2019 and 2017 with an nRMSE of 16 and 19% and d value of 0.95 and 0.97, respectively, whereas in 2018, the nRMSE and d values were 36% and 0.93, respectively (Fig. 3). The top weight simulation resulted in nRMSE was 9% and d value was 0.99 in the year 2019. The nRMSE (21%) and d value (0.97) were for 2017, and the nRMSE (17%) and d values (0.98) were for 2018. The lower values for nRMSE and higher d-values close to one revealed that the model simulated the LAI and top weight quite well.

The evaluation statistics of the simulated and measured leaf area index (LAI) at the grain filling stage and the top weight at the final harvest are presented in Table 3. The simulated LAI and top weight values were in very good agreement with the measured LAI and top weight under optimum plant density (225 plants m^−2^) and sufficient nitrogen fertilizer (200 kg ha^−1^ N). The RMSE, nRMSE, E, and d values of the simulated and measured LAI (0.57 m^2^m^−2^, 11.18, 0.86, and 0.95, respectively) and top weight (1.5 10^3^ kg ha^−1^, 12.31%, 0.92, and 0.96, respectively) (Table 3). This result indicates that the calibration of the CERES-Wheat model exhibited acceptable levels.

### Wheat grain yield and harvest index

The values of the evaluation statistics of simulated and measured grain yield (GY) and harvest index (HI) at the final harvest are presented in Table 3. The model accurately simulated GY and HI, with a simulated/measured GY of 4346/4373 kg ha^−1^ and HI of 0.38/0.37% under normal plant density (225 kg ha^−1^) and nitrogen fertilizer (200 kg ha^−1^) supply. The CERES-Wheat model slightly underestimated the grain yield and over-predicted the harvest index compared with the measured grain yield and harvest index (Table 3). The statistical indicators also indicated good agreement between the simulated and measured values for both GY and HI, with RMSE, nRMSE, E, and d values of 500 kg ha^−1^, 11.50%, 0.98, 0.89, and 0.02%, 5.40%, 0.94, and 0.98, respectively (Table 3). The normalized RMSE values were considered to indicate ‘good agreement’ when ≤ 15%, while the d-index was considered ‘excellent’ when > 0.9^39^. Similarly, the statistical indicators confirmed that the model predicted wheat grain yield and harvest index reasonably well, with respective nRMSE and d-index of 7% and 0.82^38,40^. Overall, the comparison between the measured and simulated data shows a reasonably good calibration of the CERES-Wheat model for anthesis date, maturity date, in-season LAI and top weight, grain yield, top weight, and harvest index at the final harvest. Thus, these results confirmed that the calibration CERES-Wheat model was suitable for simulating phenology, LAI, top weight, grain yield, and harvest index for long-term prediction.

### Model verification

**Fig. 5:**
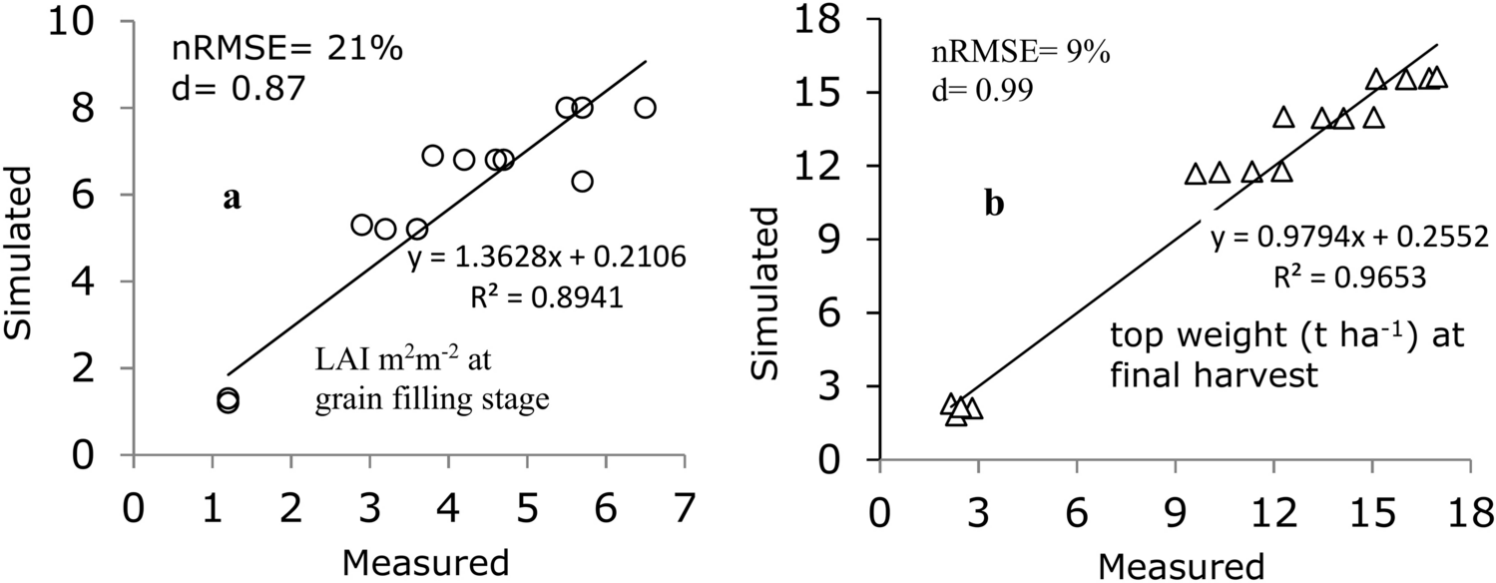
Comparison between simulated and measured leaf area index (**a**) at grain filling stage and top weight (**b**) at final harvest for model evaluation data obtained from the 2020 cropping season.

The model was further verified with the experimental data collected during 2020, under four plant densities (PD_1_, PD_2_, PD_3_ and PD_4_ plants m^−2^) and four nitrogen (N_0,_ N_1_, N_2_ and N_3_ kg ha^−1^N) rates. The CERES-Wheat model slightly overpredicted the LAI (5.1 m^2^ m^−2^) compared to the measured LAI (3.4 m^2^ m^−2^). The simulated LAI at the grain filling stage under rain-fed condition was comparatively less satisfactory, with an nRMSE (21%) and d index (0.82) (Fig. 5a). A possible reason might be that low soil moisture availability at the grain-filling stage resulted in a higher difference between the simulated and measured LAI values. The simulated and measured top weights at the final harvest matched well, with nRMSE and d values of 9% and 0.99, respectively (Fig. 5b). This result suggests that the top weight was better than the LAI variable, which is consistent with the results of the previous studies by^40,41^.

**Fig. 6:**
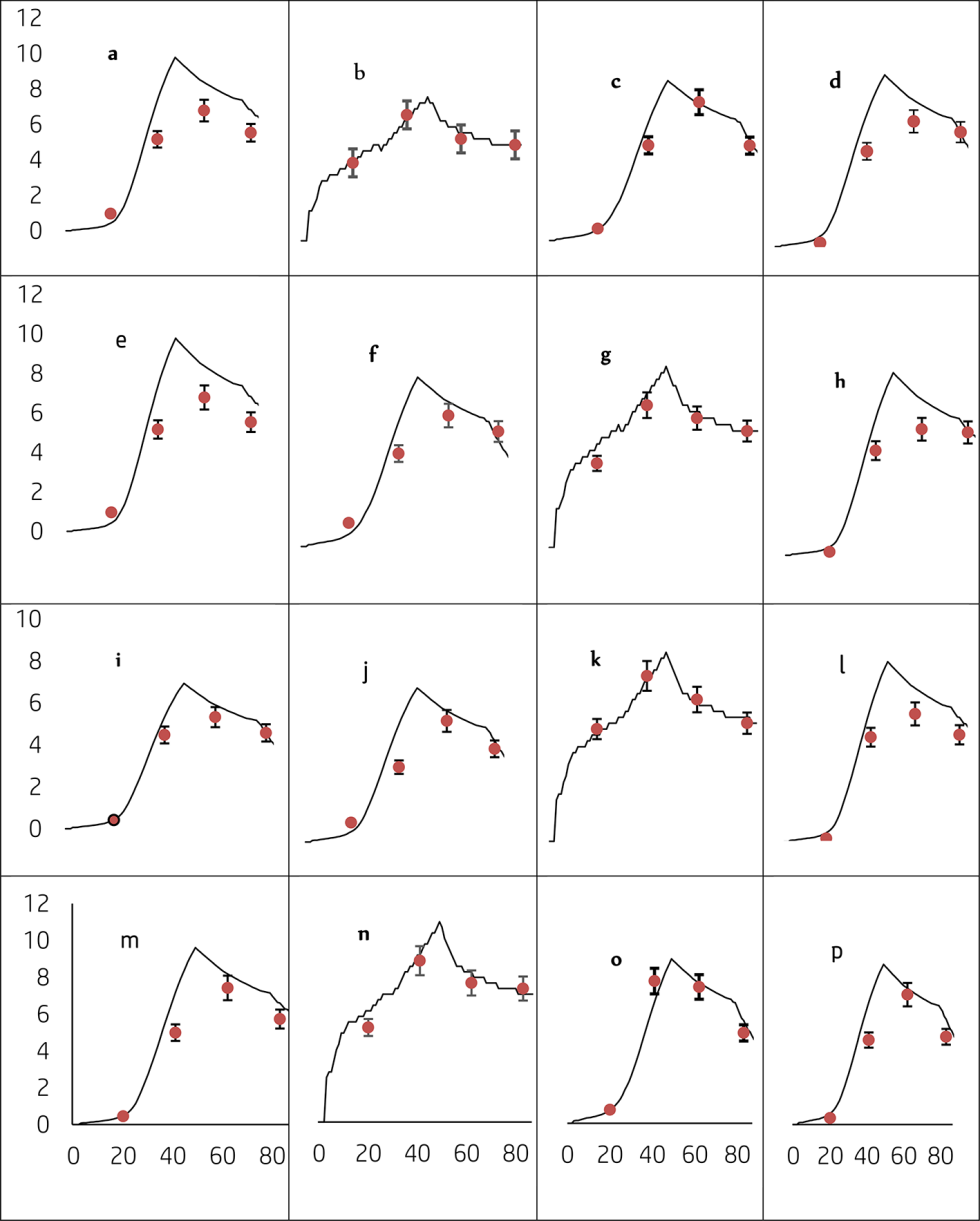
Simulated (continuous line) measured (circular with error bar symbol) leaf area index of durum wheat variety Utuba under four plant density and N fertilizer application rates, i.e., PD_1_ + N_0_ (**a**), PD_1_ + N_100_ (**b**), PD_1_ + N_150_ (**c**), PD_1_ + N_200_ (**d**), PD_2_ + N_0_ (**e**), PD_2_ + N_100_ (**f**), PD_2_ + N_150_ (**g**), PD_2_ + N_200_ (**h**), PD_3_ + N_0_ (**i**), PD_3_ + N_100_ (**j**), PD_3_ + N_150_ (**k**), PD_3_ + N_200_ (**l**), PD_4_ + N_0_ (**m**), PD_4_ + N_100_ (**n**), PD_4_ + N_150_ (**o**), and PD_4_ + N_200_ (**p**) under rain-feed condition at Memir Hager 2020 for model evaluation.

**Fig. 7:**
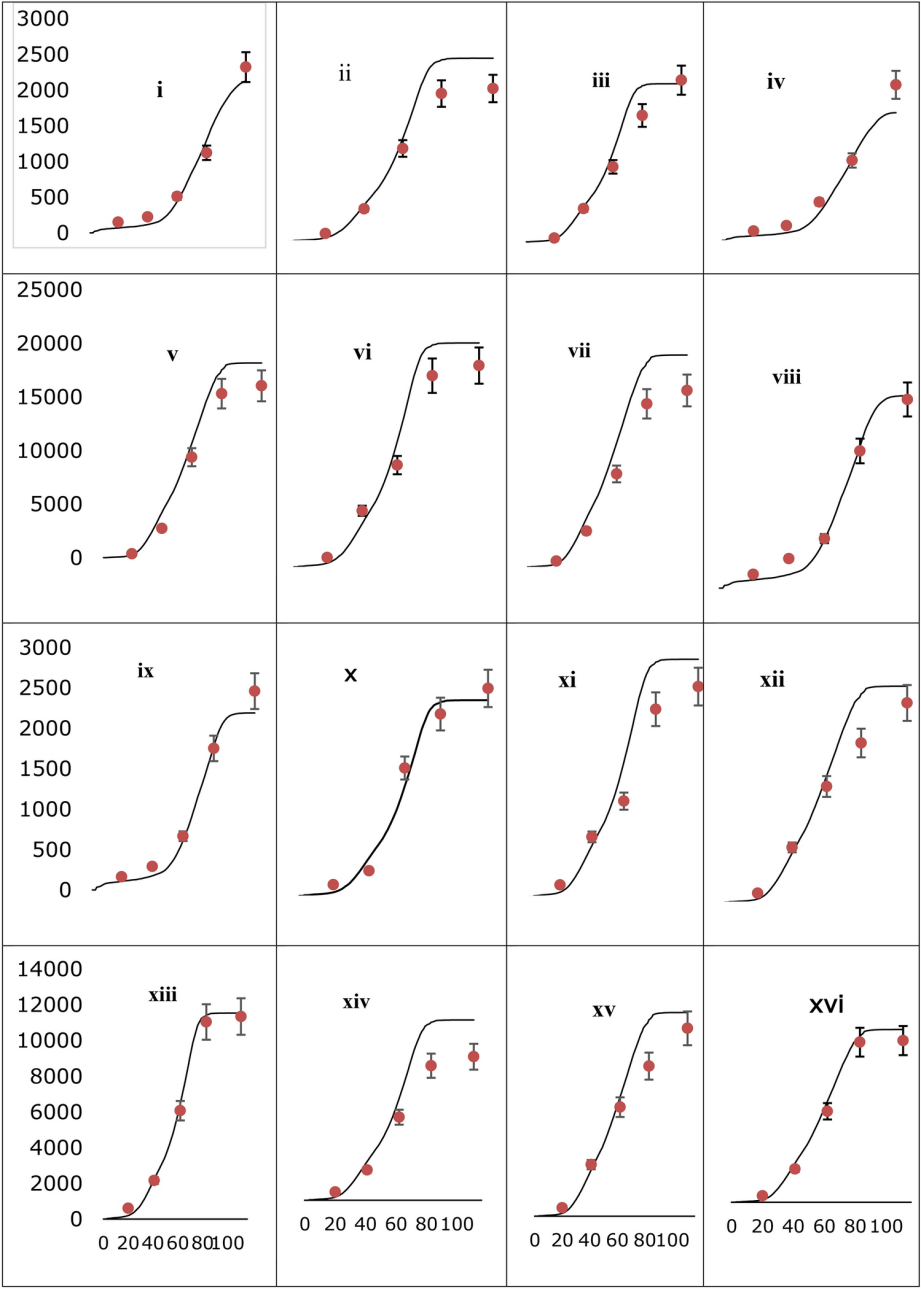
Simulated (continuous line) measured (circular with error bar symbol) top weight of durum wheat variety Utuba under four plant density and N fertilizer application rates, i.e., PD_1_ + N_0_ (i), PD_1_ + N_100_ (ii), PD_1_ + N_150_ (iii), PD_1_ + N_200_ (iv), PD_2_ + N_0_ (v), PD_2_ + N_100_ (vi), PD_2_ + N_150_ (vii), PD_2_ + N_200_ (viii), PD_3_ + N_0_ (ix), PD_3_ + N_100_ (x), PD_3_ + N_150_ (xi), PD_3_ + N_200_ (xii), PD_4_ + N_0_ (xiii), PD_4_ + N_100_ (xiv), PD_4_ + N_150_ (xv), and PD_4_ + N_200_ (xvi) under rain-feed condition at Memir Hager 2020 for model evaluation.

Time-series simulated and measured LAI and top weight of durum wheat are set out in Table 4. The simulated trends of LAI and tops weight at growth stages (GS24 (tillering), GS40 (booting), GS60 (flowering), and GS80 (grain filling)) for different plant density (PD_1_, PD_2_, PD_3,_ and PD_4_) plants m^−2^ and nitrogen rates (N_0,_ N_1_, N_2,_ and N_3_ kg ha^−1^) are presented in Fig. LAI (a-p) (Fig. 6) and top weight (i-xv) (Fig. 7). The trend observed between the simulated and measured of LAI showed a good agreement with those of measured LAI. The statistical values of nRMSE and d-index between simulated and measured LAI for different treatment combinations ranged from 10 to 31 and 0.80 to 0.99 in respective order (Table 4). Similarly, the trend between simulated and measured top weight was also closely linked with nRMSE and d-value which ranges from 7 to 37% and 0.93 to 0.98, respectively (Table 4). Most treatment combinations except PD_1_ + N_2_, PD_3_ + N_2,_ and PD_4_ + N_2_ showed low values for nRMSE and high d-values compared to the nRMSE value of 25, 31.6 and 29.6% for LAI and 30.3, 30.5, and 37.6% for tops weight, respectively. Higher nRMSE indicated that the model predicted LAI and top weight poorly. While the other treatment combinations indicated low nRMSE and high d-values was close related and precisely predict LAI and top weight. Overall, simulated and measured LAI and top weight were fair agreement, which showed that the calibrated DSSAT-CERES-wheat model could simulate the LAI and top weight of durum wheat in Ethiopian conditions.

**Table 4:** The root means square error (RMSE), normalized RMSE (NRMSE), model efficiency (E), and d-value leaf area index (LAI), and top weight of durum wheat as affected by plant density and N fertilizer rates in 2020 cropping season.

Leaf area index (LAI)					Tops weight			
Treatment	RMSE (m^2^/m^2^)	nRMSE (%)	E	d-index	RMSE (kg/ha)	nRMSE (%)	E	d-index
175 plant m^−2^								
0 kg ha^−1^ N	0.02	13.5	0.86	0.80	98.2	19.6	0.97	0.97
100 kg ha^−1^ N	0.41	14.4	0.94	0.98	1237.4	28.1	0.98	0.96
150 kg ha^−1^ N	0.88	25.5	0.90	0.95	1575.9	30.3	0.98	0.95
200 kg ha^−1^ N	0.69	14.9	0.94	0.98	494.5	7.1	0.97	0.98
225 plant m^−2^								
0 kg ha N	0.02	19.4	0.76	0.91	173.1	26.9	0.97	0.95
100 kg ha^−1^ N	0.62	21.9	0.87	0.95	1203.9	26.1	0.97	0.96
150 kg ha^−1^ N	0.55	15.0	0.96	0.98	602.6	10.3	0.98	0.98
200 kg ha^−1^ N	0.75	15.7	0.92	0.98	960.4	15.1	0.98	0.97
275 plant m^−2^								
0 kg ha^−1^ N	0.03	10.9	0.99	0.88	129.6	19.6	0.98	0.97
100 kg ha^−1^ N	0.55	17.9	0.88	0.97	496.2	10.0	0.98	0.98
150 kg ha^−1^ N	1.09	31.6	0.81	0.92	1577.2	30.5	0.98	0.95
200 kg ha^−1^ N	0.50	10.9	0.97	0.99	1127.0	15.9	0.96	0.97
325 plant m^−2^								
0 kg ha^−1^ N	0.03	11.3	0.65	0.86	130.7	18.2	0.97	0.97
100 kg ha^−1^ N	0.31	10.0	0.97	0.99	812.8	15.9	0.97	0.97
150 kg ha^−1^ N	0.98	29.6	0.90	0.94	1948.6	37.6	0.93	0.93
200 kg ha^−1^ N	0.84	18.1	0.90	0.97	768.5	10.1	0.98	0.98

### Grain yield

**Fig. 8:**
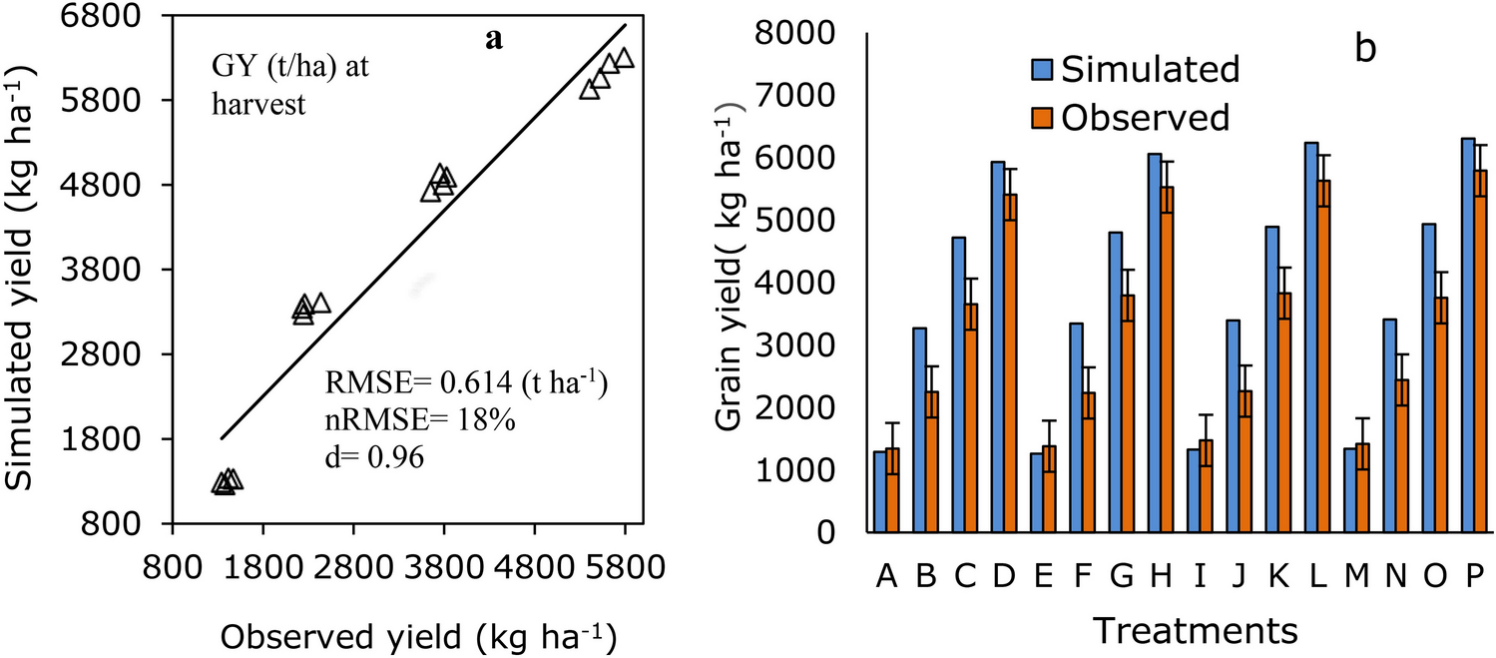
Relationships between the simulated and measured grain yield (**a**). Comparison of simulated and measured grain yield of durum wheat under four planting density and four N fertilizer rates (**b**). Note: PD_1_ + N_0_ (A), PD_1_ + N_100_ (B), PD_1_ + N_150_ (C), PD_1_ + N_200_ (D), PD_2_ + N_0_ (E), PD_2_ + N_100_ (F), PD_2_ + N_150_ (G), PD_2_ + N_200_ (H), PD_3_ + N_0_ (I), PD_3_ + N_100_ (J), PD_3_ + N_150_ (K), PD_3_ + N_200_ (L), PD_4_ + N_0_ (M), PD_4_ + N_100_ (N), PD_4_ + N_150_ (O), and PD_4_ + N_200_ (P). Vertical bars are standard deviations of measurements and simulations for model evaluation.

The simulated grain yields (GY) are like the measured grain yields (Fig. 8a) with RMSE, nRMSE, and d values of 0.614 t ha^−1^, 18%, and 0.96 for model evaluation (Fig. 8a). The simulated trends in grain yield under different planting densities and N fertilizer rates were in good agreement with those of the measured GY. However, the model slightly overestimated the grain yield compared to the measured yield in all treatment combinations, except for all plant densities under the no N fertilizer treatment (Fig. 8b). The accumulation of high biomass growth observed at early growth in good rain followed by a dry period after termination of rain probably enhanced rapid, quick senescence, reduced sink source relation, grain formation, and all contributed to the final yield reduction^22^. In contrast, the model predicted grain yield slightly lower than the measured grain yield in treatment combinations for no N fertilizer across all plant populations (Fig. 8b). Similarly, in the present study the grain yield predication obtained compared to previous the CERES-Wheat model showed poor performance using nitrogen than no N^42,43^. In Durum wheat, the combination of PD_4_ + N_3_ had greater grain yield than the no N treatment an across all plant densities (Fig. 8b). However, no significant difference was observed between PD_2_ + N_3_ and PD_4_ + N_3_ treatment combinations (Fig. 8b). The GY of durum wheat under different plant densities and N fertilizers varied compared to different treatment combinations for simulation/measured GY which ranged from 1260/1380 under treatment combinations of PD_4_ + N_0_ to 6304/5790 kg ha^−1^ in PD_4_ + N_4_. In general, good agreement between simulated and measured GY values showed that the calibrated CERES-Wheat model could simulate GY of durum wheat very well in the study.

### Model applications

**Fig. 9:**
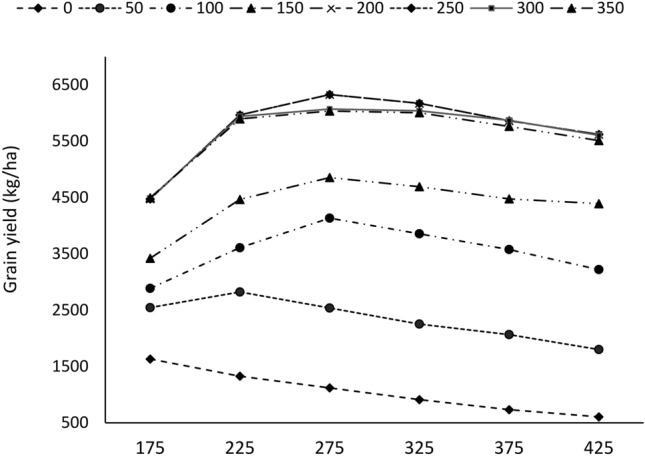
37-year average simulated grain yield response to plant density and nitrogen fertilizer rates under rainfed conditions.

The analysis to determine the optimum plant density and nitrogen fertilizer rate for durum wheat cultivation in the central highlands of Ethiopia was conducted using the CRESE-wheat model. The CERES-Wheat model was used to simulate grain yield for durum wheat in 48 different treatment combinations, with six plant densities ranged from 175 to 425 plants m^−2^ and eight nitrogen fertilizer rates ranging from 0 (control) to 350 kg ha^−1^ using 37 (1985 to 2022) years of historical daily weather data under the rain-fed farming system. The 37-year average grain yield increased with increasing nitrogen fertilizer rates from 100 to 250 kg ha^−1^ N as the plant density increased from 175 to 275 plants m^−2^ (Fig. 9). Under 0 (control) kg ha^−1^ N fertilization, the 37-year average grain yield decreased from 1632 to 765 kg ha^−1^ as plant density increased from 175 to 425 plants m^−2^ (Fig. 9). These results indicate that, under no N fertilization, plant density had a negative effect on grain yield in populations above 175 plants m^−2^. Under high plant density, soil nutrient depletion was high and exacerbated when the soil was incapable of supplying nutrients during crop growth, resulting in decreased grain yield.

From the simulated results, the highest (6328 kg ha^−1^) grain yield was predicted at a nitrogen rate of 250 kg ha^−1^ interaction with 275 plants m^−2^ followed by 200 kg ha⁻^1^ with 275 plants m⁻^2^ (6071 kg/ha), compared to all combinations of nitrogen rate and plant density (Fig. 9). No further increases in the 37-year averaged grain yield were observed when the plant density exceeded 275 plants m^−2^ under all nitrogen fertilizer rates. The 37-year simulated average yield increased with increasing plant density and nitrogen fertilizer until a critical point (250 kg ha^−1^ N and 275 plants m^−2^), and then declined with further increases in both nitrogen and plant density. Thus, a nitrogen rate of 250 kg ha^−1^ and a plant density of 275 plants m^−2^ maximized the grain yield for the study site under rain-fed conditions. Durum wheat yield increased when plant density and nitrogen fertilizer increased until a critical point, mainly due to better uptake of resources, especially nitrogen and soil water, while maintaining an optimum spike density. However, as nitrogen nutrient and plant density increased beyond these critical points (250 kg ha^−1^ and 275 plants m^−2^), the yield declined mainly because of increased competition for resources, especially soil water and solar radiation. Under rainfed conditions (water-limited), crop yield is also limited by soil water and solar radiation. Nitrogen nutrients and plant density increased beyond the critical point, and the photosynthetic characteristics of the plant declined, resulting in lower crop photosynthetic assimilation and yield productivity per plant, which might explain the decrease in durum wheat yield observed in this simulated scenario at high nitrogen and plant density^13^. ^16^ reported that plant density exceeded the critical point, and solar radiation became the yield-limiting factor, especially under high-nitrogen conditions. The simulated results indicated that pursuing high nitrogen fertilizer and plant density are not desirable strategies in the rainfed farming system, whereas the relatively optimum nitrogen and plant density may be more conducive to the effective use of resources in the rainfed farming system for yield sustainability.

### Agronomic use efficiency

**Fig. 10:**
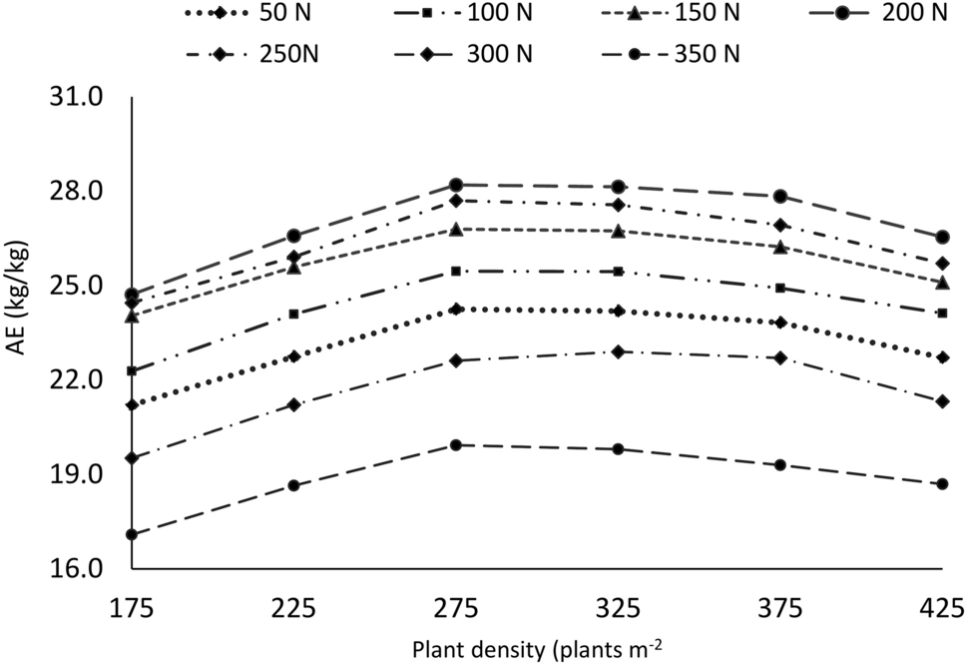
37-year average simulated agronomic use efficiency response to plant density and nitrogen fertilizer rates under rainfed condition.

The agronomic use efficiency (AUE) and partial factor productivity (PFP) in response to plant density and nitrogen fertilizer rate are shown in Fig. 10. The simulation results showed that the response of AE to plant density and nitrogen rate was linear. For all nitrogen fertilizer rates except the control (zero), the AUE of durum wheat increased as plant density increased. However, when the plant density exceeded 275 plants m^−2^ along with the nitrogen rate, the AUE tended to decrease. The rate of 350 kg N ha^−1^ at all plant densities resulted in the lowest AUE; however, the dominant AUE was 200 kg ha^−1^ N at all plant densities (Fig. 10). The treatment combination of 200 kg ha^−1^ and 275 plants m^−2^ followed by 250 kg ha^−1^ and 275 plants m^−2^ was given the highest AUE among the 48 simulated scenarios. The rate of 200 kg ha^−1^ and under all plant densities produced AUE ranging from 24.7 to 28.2% (average 27%) and 250 kg ha^−1^ and under all plant densities, AUE ranged from 24.5 to 27.7 (average 26.4). Owing to this finding, increased two-level plant density from a lower level (175 plants m^−2^) under a 200 kg ha^−1^ N rate can enhance nitrogen use efficiency. This is because increasing plant density enhances nitrogen and nutrient uptake and utilization efficiency by the roots of durum wheat and accelerates the transfer of nitrogen nutrients from the roots to stems and leaves^43^, cumulatively increasing nitrogen use efficiency^16,44^. Hence, increased durum wheat plant density, along with nitrogen fertilizers up to a certain level, is important for reducing nitrogen fertilizer losses and environmental risks while increasing grain yield^45^. Once the optimum plant density and nitrogen fertilizer rates were exceeded, both grain yield and AUE decreased (Fig. 10).

### Economic analysis

**Fig. 11:**
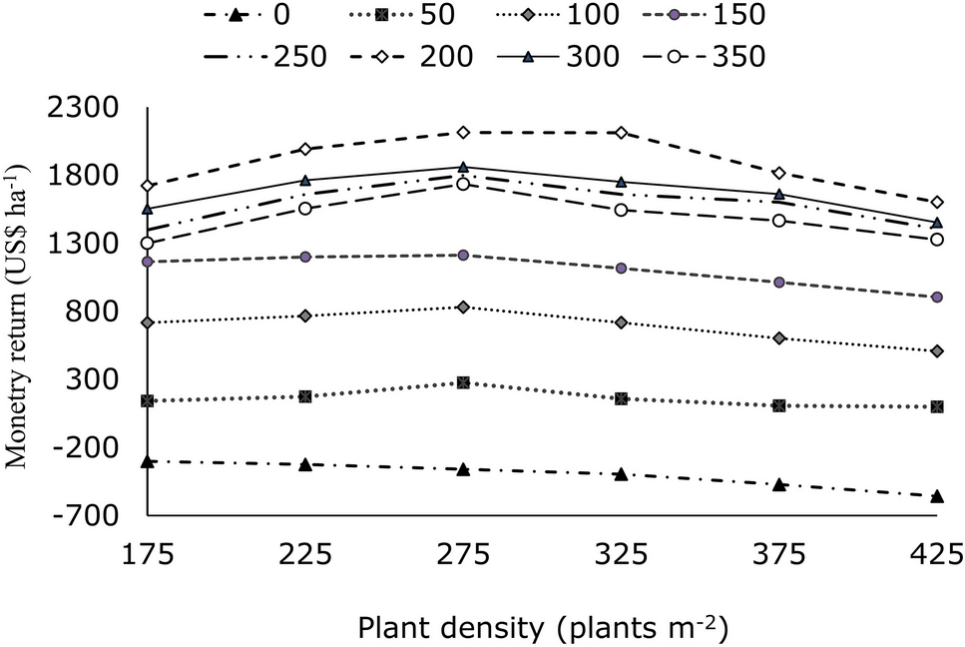
The 37-year average simulated monetary return US$ ha^−1^ variable plant density and N application rates.

The monetary return of US$ ha^−1^ for all 48 scenarios is presented in Fig. 11. Strategic analysis was used to identify the best strategic treatment combinations for sustainable durum wheat production. In the simulation scenarios, at all plant densities without nitrogen fertilizer application, the 37-year monetary returns indicated a negative profit (Fig. 11). However, at all other nitrogen rates, monetary returns increased and became positive as plant density increased from 175 to 275 plants m⁻^2^. Beyond 275 plants m⁻^2^, monetary returns began to decline with further increases in plant density (Fig. 11). The result of the strategic analysis of monetary returns $ha^−1^ (Fig. 11) showed that the treatment combination of 200 kg ha^−1^ and 275 plants m^−2^ was the highest among the 48 simulated scenarios. These results are consistent with those of another study that applied the CERES-wheat model^22^. This study, which was conducted in the northern part of Ethiopia, showed that the treatment in which 160 kg ha^−1^ was applied was dominant compared to other rates. However, their study was conducted under irrigation conditions using bread wheat cultivars and environments than in our study, and the lowest N application rate was 160 kg ha^−1^.

## Conclusion

The CERES-Wheat model was calibrated, evaluated, and used to determine the optimum plant density and nitrogen fertilizer rate for durum wheat cultivation under rainfed condition. The results for model calibration under optimum input treatment experiments and evaluation under variable plant density and N rates showed that the model between simulated and measured values for phenology, leaf area index, top weight, grain yield, and harvest index were in good agreement. However, the model slightly overestimated the predicted grain yield compared to the measured yield in the N treatment compared to the unfertilized treatment at all plant densities. Simulation scenarios were tested using 37 years of historical weather data, and seasonal analysis showed how to better optimize plant density and N fertilizer rate to optimize yield, nitrogen use, and economic return. Based on the seasonal analysis of a 37-year simulation, the optimal combination of planting density and nitrogen management was found to be 275 plants m^−2^ with 200 kg ha^−1^ N. The simulation results suggested that a plant density of 275 plants m^−2^ with an N application of 200 kg ha^−1^ increased grain yield, improved nitrogen use, and produced the highest economic return while minimizing environmental risk under rainfed conditions. The approach used and the results generated in this study can help develop the best management strategies for advancing crop yield and nitrogen use with the highest economic return while reducing the negative effect on the environment. However, the model was tested and simulated under rainfed conditions. It is advisable to undertake future research to test and simulate the model under irrigation conditions for further application of the model.

## Data Availability

The datasets used and/or analyzed during the current study are available from the corresponding author upon reasonable request.
